# What Works Well for People With Dementia and Their Supporters From South Asian, African and Caribbean Communities in the UK: A Narrative Synthesis Systematic Review and Expert Consultations

**DOI:** 10.1002/gps.70047

**Published:** 2025-02-26

**Authors:** Orii McDermott, Thea Sobers, Naaheed Mukadam, Abigail Rebecca Lee, Martin Orrell

**Affiliations:** ^1^ School of Medicine University of Nottingham Nottingham UK; ^2^ Dementia UK London UK; ^3^ Division of Psychiatry University College London London UK; ^4^ Neurology Research Research & Innovation Nottingham University Hospitals NHS Trust Nottingham UK; ^5^ Institute of Mental Health Nottinghamshire Healthcare NHS Foundation Trust Nottingham UK

**Keywords:** African and Caribbean, care, dementia, preferred support, South Asian, systematic review

## Abstract

**Objectives:**

This review aims to synthesise the evidence regarding the use and provision of dementia services and support for people with dementia and/or supporters from South Asian, African or Caribbean backgrounds living in the UK.

**Methods:**

A narrative synthesis systematic review of the original research articles published up to April 2024 was conducted. A lay summary of the initial review findings was evaluated by experts‐by‐experience (*n* = 15) for scrutiny and to enable further discussions, to produce key recommendations for further developing dementia services.

**Results:**

A total of 18 studies (16 qualitative and 2 mixed methods studies) met the full inclusion criteria and were included in the review. The review findings and experts‐by‐experience consultations highlighted that: (1) dementia is not openly discussed or disclosed within many diverse ethnic communities. This can lead to family carers and people with dementia feeling isolated and unsupported. (2) Mainstream dementia support services and hospitals often do not meet diverse communities' cultural and religious needs, and (3) home‐based care supported by external care agencies can be helpful but ensuring consistency of care staff in their culturally appropriate care can be extremely difficult to ensure.

**Conclusions:**

Encouraging South Asian, African and Caribbean communities to increase their dementia knowledge is important. However, mainstream dementia support services also need to incorporate their cultural and religious essentials into care packages to encourage their help seeking behaviours and tackle dementia stigma. Collaborative service developments between the diverse communities, Health and Social Care providers and policy makers are essential to ensure equitable and culturally appropriate dementia care for diverse community members in the future.

**Summary**

- Dementia is not openly discussed or disclosed within many diverse ethnic communities. This can lead to family carers and people with dementia feeling extremely isolated. Religious leaders can have an important role in normalising talking about dementia and reducing perceived stigma attached to dementia and wider mental health.
- Family orientated support was considered most culturally appropriate by many. Home‐based support from external agencies could benefit family carers, but agency carers should have a full understanding of clients' religious and cultural needs particularly when providing personal care and supporting food preparation.
- Mainstream dementia care organisations try to be inclusive by welcoming diverse communities into their activities or day services. However, many members from South Asian, African and Caribbean communities have experienced mainstream services often do not provide culturally appropriate activities or food, and therefore, do not think these services would be helpful to them.
- Diverse communities can help to educate health and social care professionals if appropriate platforms are available to share essential information on their cultural norms and religious practices to provide appropriate dementia care.

## 1 Introduction

Around 25,000 people living with dementia in the UK are from ethnic minority communities [1]. People from diverse ethnic backgrounds often present to diagnostic and therapeutic services at a later stage of their dementia [2] and there are barriers to seeking appropriate support [3, 4, 5]. Researchers have sought to understand help‐seeking behaviour of people from diverse ethnic groups [2, 6]. However, this is a complex task as individuals' acceptance and understanding of dementia and wider mental health issues are deeply rooted in their embedded cultural, religious, societal and personal values. As highlighted by our public research collaborators, if one comes from a cultural background where mental health issues are not widely discussed, it can be challenging for someone experiencing dementia symptoms to seek external support beyond one's immediate family members.

The 2021 Census [7] identified that people from Asian ethnic groups made up the second largest percentage of the England and Wales population (9.3%), followed by black ethnic groups (4%). This review will therefore focus on people from South Asian, African and Caribbean backgrounds living in the UK. Whilst the diversity of cultural and religious heritages exists amongst both ‘South Asian’ and ‘African and Caribbean’ ethnic groups, we will use these two terms broadly for this review but will refer to specific ethnic subgroups where possible. In this review, ‘dementia services’ will be defined as covering the diagnostic process and post‐diagnostic support, including both hospital and community‐based services.

## 2 Objectives

This review aims to explore the evidence regarding the use and provision of dementia services and community support for people with dementia and/or supporters (families, friends, community members) from South Asian, African and Caribbean backgrounds in the UK. We aim to identify the key components of facilitators (‘what works well’ for people) as well as existing barriers. With inputs from experts‐by‐experience, we aim to develop practical recommendations to improve access to support services by these community groups and to help stakeholders and policymakers in the UK to develop culturally appropriate valued‐based care provision for people with dementia from South Asian, African and Caribbean backgrounds.

## 3 Methods

A Narrative Synthesis (NS) Systematic Review was conducted since this approach allows a transparent, systematic evaluation and synthesis of both quantitative and qualitative studies [8]. It is also suitable to investigate *how* and *why* specific interventions or approaches may work for certain groups of people by using its four interactive review elements: 1. Developing a theory, 2. Developing a preliminary synthesis, 3. Exploring relationships within and between studies, and 4. Assessing the robustness of the synthesis [8, 9].

### 3.1 Development of a Review Protocol (NS Element 1)

A draft review protocol was developed in accordance with the PRISMA guideline [10]; based on the initial team discussion and electronic searches to ensure no similar reviews were currently being undertaken. To strengthen the validity of the third NS element (exploration of the study findings) and the fourth NS element (synthesis of the findings), we: (1) produced an initial review summary report and shared with experts‐by‐experience, then (2) conducted consultation sessions to seek experts' views on the initial findings and obtain further insights to integrate into this review's findings and future recommendations. The finalised protocol was forwarded to the Information Specialist in the Nottinghamshire Healthcare NHS Foundation Trust, who conducted electronic database searches.

### 3.2 Search Strategy

Stage 1:

The following search engines were used to find studies up to 20 December 2022:

- Cochrane Central Register of Controlled Trials (CENTRAL) Issue 11, 2022, in the Cochrane Library;
- MEDLINE Ovid (from 1946 onwards);
- APA PsycInfo ProQuest (from 1806 onwards);
- APA PsycArticles ProQuest (from 1800 onwards);
- Web of Science (Clarivate) (from 1900 onwards);
- Scopus Elsevier (from 1788 onwards).

The results of the searches were combined and deduplicated in a database. The search strategies are available in Supplemental Material S1 in Supporting Information S1.

Stage 2:

The original search strategy was re‐run up to 23 April 2024 and an entry date filter was added.

### 3.3 Inclusion Criteria

- The study participant population are adults aged 18 or over who have a diagnosis of dementia, and/or supporters of people with dementia, who self‐identify from South Asian, African or Caribbean communities and live in the UK
- Original research data on the participants' use of/access to dementia services or community support for people with dementia is included
- Peer‐reviewed original journal articles published up to April 2024

### 3.4 Exclusion Criteria

- Systematic reviews, discussion and theoretical articles without original research data, case reports, project reports, opinion pieces, book chapters, conference abstracts, thesis
- Articles without evidence of peer‐review
- Study participants do not include people with dementia and/or their supporters from South Asian, African or Caribbean communities living in the UK
- Studies that do not meet the minimum Quality Assessment standard (as defined below)

All study designs (quantitative, qualitative, mixed methods, survey) were eligible provided they met the full inclusion criteria.

### 3.5 Study Screening and Selection

Study screening and selection was managed using Rayyan Software. Titles and abstracts were screened initially by one reviewer (TS). All articles that clearly did not meet the full inclusion criteria were excluded. Full text articles were obtained for the remaining articles that required closer investigation. Reference lists of provisionally included studies were hand searched. Full text screening and quality assessment of these articles were completed independently by two reviewers (OM and TS) first. Discrepancies were resolved through discussion.

### 3.6 Quality Assessment

All studies that met the initial inclusion criteria used qualitative and mixed method approaches. The Critical Appraisal Skills Programme (CASP) Qualitative Checklist [11] and the modified COREQ Checklist [12] were used for quality assessment of the studies. The details of Quality Assessment process are provided in Supplemental Material S2 in Supporting Information S1.

### 3.7 Data Extraction (NS Element 2)

A data extraction form was created to extract data for the key domains: including study characteristics, population characteristics, service details (if applicable), key findings, and quality of study.

### 3.8 Production of Lay Summary Report (NS Element 2, 3)

Following the initial data analyses, a five‐page lay summary report was produced, consisting of five sections: (1) What is a systematic review?, (2) Why are we conducting this review?, (3) How we selected the research studies for our review, (4) Summary of what we found out from the articles included in the review, with two subsections: (a) What research participants from the South Asian community reported, (b) What research participants from the African and Caribbean community reported, and (5) What happens during our focus group meeting? The finalised lay summary report was forwarded to the Centre for Ethnic Health Research team (hereafter ‘the Centre’) in Leicester, who agreed to distribute the report to focus group and interview participants at least 2 weeks before the meeting date.

### 3.9 Expert Consultations: Focus Groups and a Family Interview (NS Elements 1, 2, 3)

Two focus groups (one with South Asian supporters, another with African and Caribbean supporters), and a Gujarati‐speaking family interview with a translator were conducted between July and August 2023. All participants were recruited by the Centre and were already known to the Centre's team. OM and TS, with support from the Centre's community engagement officers, conducted the consultation sessions. With permission from the participants, the sessions were audio recorded. No personally identifiable information was collected by the review team, but the participants agreed to provide their demographic information anonymously using an online form. No institutional ethical approval was sought to conduct this work, since all public members were asked to provide their opinions on the current dementia care services available to them (public consultations) and were not approached as research participants. To increase transparency of how contributions during focus groups and the interviews would be used for the purpose of this review, the research team produced a written information sheet and obtained consent from all participants.

### 3.10 Data Analysis of the Expert Consultations (NS Elements 1, 3)

The researchers immediately made individual notes following each session and held meetings to discuss the key outcomes. Audio recordings were transferred to secure university computers. TS produced transcriptions of all sessions, only omitting transcriptions of conversations not directly related to the contents of this review. The researchers read through the transcriptions individually and highlighted the key discussion outcomes from each session, comparing notes. They then held a meeting to agree on the key components from the expert consultations to be included in the review.

### 3.11 Data Synthesis (NS Elements 1, 4)

The draft data analysis and synthesis document was shared with NM, AL and MO to validate the initial interpretation of the review and expert consultation data. Their feedback was incorporated into further development of the Discussion section and refinement of recommendations.

## 4 Results

**FIGURE 1:**
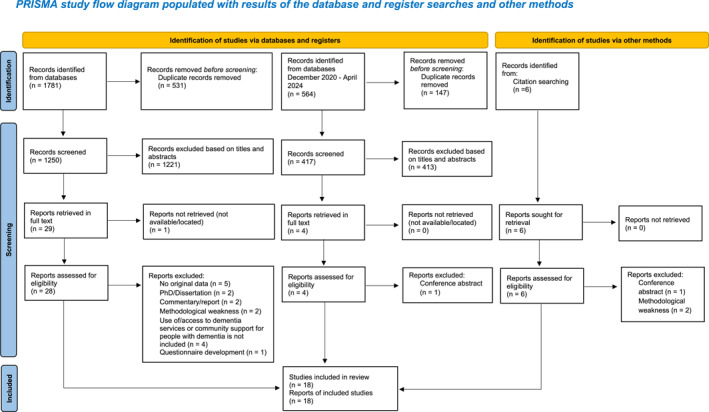
Prisma diagram.

The original electronic searches yielded 1781 articles. After removing duplicates, 1250 articles were screened for eligibility based on title and abstract. Of these, 1221 articles were excluded, and the full text of 28 of the remaining 29 potential articles were obtained. Hand searching the reference lists of these full‐text articles yielded an additional 6 papers that were included and obtained in full text. A second electronic search identified 564 articles. Following duplicate removal, title and abstract screening of 417 articles resulted in the exclusion of 413 articles. The full text of 4 articles were obtained for further screening. A total of 38 articles were assessed for eligibility, and 20 (16 from initial search, 3 from citation searching, 1 from second search) were excluded for the following reasons: no original data, dissertation/thesis, commentary/report, use of/access to dementia services or community support for people with dementia not being included, questionnaire development, methodological weakness and conference abstract. A total of 18 (16 qualitative studies and 2 mixed‐method studies) met the full inclusion and the Quality Standard and were included in this review (Figure 1).

Table 1 provides a summary of study characteristics.

**TABLE 1:** Summary of study characteristics.

Study (year)	Aims/focus of study	Study type	Data collection method	Data analysis method	n. of study participants
Baghirathan (2020)	Experience of dementia care amongst Bristol BAME communities	Qualitative	Interviews (27) Focus groups (8)	Grounded theory approach	103
Berwald (2016)	Barriers to help‐seeking for memory problems within UK black African and Caribbean communities	Qualitative	Focus groups (8) Interviews (3)	Thematic analysis	50
Brown (2021)	Memory assessment services' approach to assessing and managing minority ethnic memory issues	Survey	Online survey	(a) Quantitative data grouped into question constructs and data summarised by calculating percentages of responses, (b) qualitative data organised by common themes	20 memory assessment services
Hossain (2019)	Perspectives, knowledge and experiences of dementia care amongst Bangladeshi family supporters in England	Qualitative	Interviews (6)	Thematic analysis	6
Hossain (2020)	Barriers to accessing mainstream dementia services among UK Bangladeshi family supporters	Qualitative	Focus groups (2) Interviews (6)	Thematic analysis	27
Hossain (2022)	Experiences and perspectives of dementia care from early detection to end of life care among South Asian supporters in the UK	Qualitative	Interviews (16)	Thematic analysis	16
James (2023)	Experiences and needs of dementia care and support among South Asian and White British communities in the UK	Qualitative	Interviews (48)	Thematic analysis	62
Kevern (2022)	Influence of religion on understanding, care and experiences of dementia within a British Pakistani Muslim community	Qualitative	Interviews (7)	Thematic analysis	7
La Fontaine (2007)	Perceptions of ageing, dementia and related mental health difficulties amongst British Punjabi Indians	Qualitative	Focus groups (8)	Thematic analysis	49
Lawrence (2008)	Attitudes, experiences and needs of black Caribbean, South Asian and White British family supporters of people living with dementia in South London	Qualitative	Interviews (32)	Grounded theory	32
Lawrence (2010)	Attitudes, experiences and beliefs of black Caribbean, South Asian and White British people with dementia living in South London	Qualitative	Interviews (30)	Grounded theory	30
Mackenzie (2006)	Support needs of family supporters of people with dementia from South Asian and Eastern European communities within the UK	Qualitative ‐stage 1—Interviews Stage 2—Support group programme Stage 3—Follow‐up interviews	Interviews (42) Field notes (number not specified)	(a) Content analysis and organisation into themes, (b) Thematic analysis and cross‐referencing with interview data	21
Mukadam (2011)	Attitudes to help‐seeking for dementia and the help‐seeking pathway between different ethnic groups	Qualitative	Interviews (18)	Thematic analysis	18
Mukadam (2015)	Barriers to and facilitators of help‐seeking for dementia among UK South Asian adults with dementia	Qualitative	Focus groups (7) Interviews (5)	Interpretative Phenomenological analysis	53
Nair (2022)	Experiences of managing eating and drinking and support among people living with dementia and their supporters from ethnic minority backgrounds	Qualitative	Interviews (17)	Thematic analysis	17
Nazir (2024)	Knowledge, awareness and barriers to help‐seeking for dementia in a Pakistani‐origin community	Qualitative	Interviews (11)	Thematic analysis	11
Parveen (2016)	Perceptions of dementia and service use among British Indian, African and Caribbean, and East and Central European communities	Qualitative/Action research	Discussion groups (19) Quiz	(a) Framework analysis, (b) Thematic analysis, (c) Frequencies and percentages were calculated for quiz data	175
Regan (2016)	Experiences using dementia services within the U.K as a South Asian Muslim male	Qualitative—Case study	Multiple data sources (17) including interviews, observations & discussions/meetings	Critical Realist grounded theory approach	1

### 4.1 Quality Assessment

Quality assessment was conducted for the 22 studies which met the initial inclusion criteria. Four qualitative studies did not meet the minimum score of 10 for Modified COREQ Checklist or minimum score of 7 for CASP Checklist. CASP scores ranged from 7–10, with a mean score of 8.9 (Table 2), while the COREQ scores ranged from 10–23 with a mean score of 15.8 (Table 3). Most studies met Quality Assessment standard but participant checking, data saturation and methodological orientation and theory was often not explicitly reported.

**TABLE 2:** CASP.

	Baghirathan, (2020)	Berwald (2016)	Hossain (2019)	Hossain (2020)	Hossain (2022)	James (2023)	Kevern (2022)	Lawrence (2008)	Lawrence (2011)	Mackenzie (2006)	Mukadam (2011)	Mukadam (2015)	Nair (2022)	Nazir (2024)	Regan (2016)	La fontaine (2007)
1. Was there a clear statement of the aims of the research?	Y	Y	Y	Y	Y	Y	Y	Y	Y	Y	Y	Y	Y	Y	Y	Y
2. Is a qualitative methodology appropriate?	Y	Y	Y	Y	Y	Y	Y	Y	Y	Y	Y	Y	Y	Y	Y	Y
3. Was the research design appropriate to address the aims of the research?	Y	Y	Y	Y	Y	Y	Y	Y	Y	Y	Y	Y	Y	Y	Y	Y
4. Was the recruitment strategy appropriate to the aims of the research?	Y	Y	Y	Y	Y	Y	Y	Y	Y	Y	Y	Y	Y	?	Y	Y
5. Was the data collected in a way that addressed the research issue?	Y	Y	Y	Y	Y	Y	Y	Y	Y	Y	Y	Y	Y	Y	Y	Y
6. Has the relationship between researcher and participants been adequately considered?	Y	?	?	N	N	?	Y	?	N	N	N	N	N	?	Y	Y
7. Have ethical issues been taken into consideration?	Y	Y	Y	Y	Y	Y	Y	?	Y	Y	Y	Y	Y	Y	Y	Y
8. Was the data analysis sufficiently rigorous?	Y	Y	Y	Y	N	Y	Y	Y	Y	?	Y	Y	Y	?	Y	Y
9. Is there a clear statement of findings?	?	Y	Y	Y	Y	Y	Y	Y	Y	Y	Y	Y	Y	Y	Y	Y
10. How valuable is the research?	V	V	V	V	V	V	V	V	V	V	V	V	V	V	V	V
Total	9	9	9	9	8	9	10	8	9	8	9	9	9	7	10	10

*Note:* Y = Yes (1), N = No (0), ? = Can't tell (0), V = Valuable (1).

**TABLE 3:** Modified COREQ Checklist Fully met = 2 Partially met = 1 Can't tell = 0 No = 0.

	Methodological orientation and theory	Sampling	Method of approach	Setting of data collection	Description of sample	Interview guide/Collection methods	Data saturation	Number of data coders	Participant checking	Quotations presented	Clarity of major themes/main findings	Data and findings consistent	Total/24 (Qualitative)	Total/22 (mixed methods)
Baghirathan (2020)	2	2	2	2	1	1	0	1	2	2	2	2	19	
Berwald (2016)	0	2	2	2	2	1	2	2	0	2	2	2	19	
Brown (2021)	n/a	1	2	2	2	2	0	0	0	1	2	2		14
Hossain (2019)	0	2	0	2	1	1	0	0	2	2	1	2	13	
Hossain (2020)	0	2	0	0	1	0	0	1	0	2	2	2	10	
Hossain (2022)	2	2	2	2	1	0	2	1	0	2	2	2	18	
James (2023)	0	2	2	2	2	1	0	2	0	2	2	2	17	
Kevern (2022)	0	2	2	2	2	0	2	2	0	2	2	2	18	
La Fontaine (2007)	0	2	1	2	1	1	0	2	0	1	1	2	13	
Lawrence (2008)	1	1	0	1	2	1	2	2	0	2	2	2	16	
Lawrence (2011)	2	2	2	2	2	1	2	2	2	2	2	2	23	
Mackenzie (2006)	0	1	2	1	1	0	0	0	0	1	2	2	10	
Mukadam (2011)	0	2	2	2	2	1	2	2	2	2	2	2	21	
Mukadam (2015)	0	2	1	2	2	2	2	2	0	2	2	2	19	
Nair (2022)	1	2	2	2	2	1	0	1	0	2	2	2	17	
Nazir (2023)	0	2	2	1	1	1	1	0	0	2	1	2	13	
Parveen (2017)	n/a	0	2	2	1	1	0	2	0	1	1	1		11
Regan (2016)	0	2	2	2	2	0	0	0	0	2	2	2	14	

*Note:* Fully met = 2, Partially met = 1, Can't tell = 0, No = 0.

### 4.2 Key Findings From the Included Studies

#### 4.2.1 Participant Characteristics

Of the 18 included studies, 10 articles included South Asian participants, [5, 13, 14, 15, 16, 17, 18, 19, 20, 21]. One study focussed on African and Caribbean participants [22] and 7 articles included both African/Caribbean and South Asian participants [2, 4, 23, 24, 25, 26, 27]. Table 4 summarises the study participants recruitment method and outcome.

**TABLE 4:** Participant recruitment criteria and methods.

Study (year)	Participants inclusion criteria	Participants ethnicity, number of M/f	Recruitment methods	Location
Baghirathan (2020)	(a) Recent or current supporters of people with dementia, or (b) people working in the BAME‐led volunteer and community service organisations.	African Caribbean (25), Chinese (31) South Asian—Muslim, Hindu, Sikh (47) 78 female & 25 male	(a) Supporters recruited from (i) BAME‐led volunteer and community service organisations or, (ii) referred by the Bristol dementia Wellbeing service (b) People working in the BAME‐led VCSOs recruited through (i) personal contacts and (ii) snowball sampling	Bristol
Berwald (2016)	(a) Black African or black Caribbean community members, (b) No prior knowledge or experience of dementia/dementia services needed	Black African (28), black Caribbean (14), black British (7) and Indo‐Caribbean (1) 30 female & 20 male	Purposive sampling and snowball sampling from community	London
Brown (2021)	Current manager or clinical lead for a memory assessment service in England or Wales	White other, Irish/Gypsy travellers, Indian, Pakistani, Bangladeshi, Chinese, other Asian, mixed ethnicity, African, Afro‐Caribbean and black British backgrounds	Services recruited using emails and telephone calls after identified through the NHS England and dementia Clinical network, Royal College of Psychiatrists and memory services national Accreditation Programme	England, Wales
Hossain (2019)	(a) Current or recent supporters of Bangladeshi person with dementia, (b) supporters who identify as Bangladeshi, (c) providing dementia support for a minimum of 6 months, no longer than 2 years ago	South Asian—Bangladeshi (6) 3 female & 3 male	Purposive sampling and snowball sampling	London, Portsmouth
Hossain (2020)	(a) Bangladeshi community members (Phase 1), or (b) Bangladeshi family supporters of people living with dementia (Phase 2)	South Asian—Bangladeshi 9 female & 12 male	Purposive sampling and snowball sampling	London, Portsmouth
Hossain (2022)	(a) Recent or current supporters of people with dementia who identify as South Asian	South Asian ‐ Indian (9), Bangladeshi (6), Pakistani (1) 7 female & 9 male	Purposive sampling and snowball sampling from community support groups, “gatekeepers” to religious institutions, care homes, day centres	West Midlands
James (2023)	(a) People with a formal diagnosis of dementia who identify as South Asian or White British, (b) family supporters of people living with dementia, (c) NHS memory clinic clinicians	PLWD—South Asian (6), White British (7) Family supporters—South Asian (11), White British/Other (13) Clinicians—African, Chinese, Indian, Pakistani, White English, White European 43 female, 19 male	Purposive sampling from memory clinics across 4 NHS trusts	London, Leicester
Kevern (2022)	(a) Muslim, from a British Pakistani background, (b) experience caring for a relative with dementia at home, (c) English speaking	South Asian—Pakistani 4 female, 3 male	(a) Opportunity sampling using researcher's contact, (b) snowball sampling using social networks and WhatsApp Muslim Women group	Midlands and North England
La Fontaine (2007)	(a) Indian community members, (b) English‐, Hindi‐ or Punjabi‐ speaking	South Asian ‐ Indian 30 female & 19 male	Convenience sampling using intermediaries through cultural centres, arts and leisure centres, gurdwaras, women's groups and colleges	Not specified in paper but the project took place in Birmingham
Lawrence (2008)	Current supporters of people with dementia who identify as black Caribbean, South Asian or White British	Black Caribbean (10), South Asian (10), White British (12) 25 female, 7 male	Purposive sampling from community mental health teams for older adults, carer services and organisations and community services	South London
Lawrence (2010)	(a) Persons with a formal diagnosis of dementia, or (b) persons attending a dementia specific day centre or group, (c) identify as black Caribbean, South Asian or White British	Black Caribbean (11), South Asian (9), White British (10) 13 male, 17 female	Purposive sampling from community mental health teams for older adults, memory clinics, day centres, community mental health programmes for minority ethnic elders	South London
Mackenzie (2006)	Current family supporters of people living with dementia from a South Asian or Eastern European community	South Asian—Pakistani (11), Indian (5) Eastern European—Polish (4), Ukrainian (1)	Purposive sampling from health and social care facilities, religious centres, community resources, community radio stations and voicemail service	Northern England
Mukadam (2011)	Supporter of someone living with dementia in London	UK White (4), South Asian (5), black: African or Caribbean (5), White Irish (1), White other (1), Asian other (1), Chinese (1) 13 female & 5 male	Purposive sampling via clinicians and the city mental health trust	London
Mukadam (2015)	(a) South Asian members of the community, (b) English or Bengali speaking	South Asian primarily from India, Pakistan, Bangladesh, Nepal and Sri Lanka	Purposive sampling from South Asian community centres and snowball sampling from community centre and personal/professional contacts	London
Nair (2022)	(a) Current on former adult supporter of someone with moderate to advanced dementia living at home, or (b) person living with early stage of dementia diagnosed more than 6 months prior and self‐identify as an ethnic minority	Indian (7), Pakistani (2) Bangladeshi (1), black Caribbean (4) Chinese (1), mixed Chinese/White (1), Other/North African (1) 11 female & 6 male	Purposive sampling from (i) GP practices, memory services using letters and leaflets, (ii) social media, previous studies, online dementia research and carer support websites using postal invites and emails, (iii) local carer organisations and dementia services via organisation member, then screened via telephone after being contacted by researcher	London
Nazir (2024)	(a) Pakistani member of the community, (b) English‐, Urdu‐ or Pothwari‐ speaking	South Asian—Pakistani 9 female, 2 male	Snowballing sampling from personal contacts and neighbourhood contacts	Stoke‐on‐Trent
Parveen (2016)	(a) People living with dementia, or (b) supporters of people living with dementia, or (c) members of the community, (d) identify as Indian, or African or Caribbean, or East and Central European	British Indian (62), African and Caribbean (50), East and Central European (63)	Advertisements in local venues	North England
Regan (2016)	South Asian person living with dementia	South Asian—Pakistani 1 male	Part of an educational project	U.K (specific location not mentioned)

### 4.3 Attitudes Towards, and Awareness of Dementia

The impact of living with dementia and associated mental health issues tend not to be widely discussed in South Asian, African or Caribbean communities or within individual families [13, 15]. The included studies widely acknowledged people from diverse ethnic backgrounds did not immediately seek mainstream healthcare support if someone in their family began to show dementia symptoms, but preferred practical support was offered or received from individuals within their close family networks [23, 25]. It appeared to be acceptable for these communities to view dementia as a brain disease rather than a mental health condition [15]. People found it is easier to seek medical help for their physical problems, with dementia often being diagnosed during appointments for another health problem [2]. This often led to the limited understanding of dementia as being a medical condition, and the lack of awareness of available dementia services and financial assistance [4, 5, 20] or available services for cognitive problems [17]. Participants from African and Caribbean communities clearly stated they ‘don't do dementia’ [22] and highlighted the risk of mental health stigma labelled as ‘madness’ or ‘craziness’ [25], then being institutionalised [23]. One study found there was ‘no stigma’ attached to dementia amongst Bangladeshi Muslim family carers [14], but this appeared to be linked to the fact that people in the community do not widely discuss dementia. Not discussing mental health may have complex, deep‐rooted cultural and religious reasons. Baghirathan summarises the reluctance to disclose dementia diagnosis as ‘fear of diminishment within own's community’ [23]. Mackenzie [16] found ‘concealment’ was a strategy to avoid rejection and to protect the person with dementia by keeping them away from other people and the dementia diagnosis being kept ‘secret’ within the family [20]. Practical support was provided by close family or community members, but not being able to talk about challenges of living with dementia sometimes led the person to feel isolated [18].

### 4.4 Supporting People Who Have Developed Dementia Symptoms

#### 4.4.1 Perspectives and Experiences of People From South Asian Communities

There is a cultural emphasis on familial care, where close family members are primarily relied upon for providing practical support [2, 26, 27]. Caregiving was viewed as a moral obligation to the family, with helping others being considered an integral part of being a good Hindu [25, 26]. As a result, there was limited use of formal services, as family support is prioritised [18, 26] and some Asian family carers felt ‘asking for professional help as a failure to fulfil their responsibilities’ [25]. However, family carers can feel burdened, stressed, isolated, and trapped, and may require additional support to alleviate their caring responsibilities [5, 24]. Female carers in particular may require home‐based respite services to reduce their caring burden [5]. Where home‐based help from external agencies were sought, there were access barriers, due to religious and cultural beliefs, as well as issues to navigate the complex UK health care system [5]. Insufficient cultural and religious knowledge among external carers regarding hygiene and personal care practices, gender preferences, cultural‐based intimate body areas, and food preparation, were highlighted [5, 13, 14, 19], with a lack of clear written care guidelines to provide culturally appropriate care [5].

As a result there was a reluctance to use mainstream respite services and day care services as the study participants felt they did not always take their cultural backgrounds, religious customs, and dietary restrictions into consideration. Cultural backgrounds and religious beliefs may directly impact food choices and dietary restrictions, which may not be easily adaptable to South Asian food and culture from the British/Western nutritional advice that is offered [27]. Additionally, language and communication barriers were frequently reported. Participants reported dementia leaflets and GP letters were not always accurately translated. Communicating with healthcare professionals could be difficult without reliable interpreters who understand cultural contexts and nuances [5, 24].

#### 4.4.2 Perspectives and Experiences of People From African and Caribbean Communities

Support from immediate families was most common and the preferred option for most African and Caribbean people. Many study participants highlighted that wider community members would help if individuals lived alone [24, 25], while people emphasised the importance of respecting one's privacy and not interfering with individuals unnecessarily is extremely important. The value of helping others has a strong cultural and religious component [25]. Individuals from African and Caribbean communities often sought psychological and spiritual support from their faith and religious institutions, such as the church [4, 22]. Community‐based institutions like churches were perceived as more accessible and engaging than mainstream dementia services [23]. Some African‐Caribbean communities have experienced a strained relationship with the healthcare system, which has resulted in a lack of trust in the system. Concerns about the lack of privacy and confidential information when using the mainstream services [22], and the potential loss of independence and fear of being institutionalised [22, 23] seemed to contribute to the community's reluctance to seek dementia support. Study participants considered day services as a source of support, but their cultural needs were often not met in these spaces [23]. Familiar food was considered most important to maintain the personal identity [26]. Finally, when using dementia services, language was also highlighted as a barrier in the Afro‐Caribbean community, as distinct languages like Creole or Patois are spoken by many older people, which may require an interpreter [23].

### 4.5 Specific Services and Types of Wider Support People Identified as Beneficial, or Not Beneficial to Them

#### 4.5.1 Care Homes

For both South Asian and African and Caribbean communities, care homes were considered a last resort due to cultural expectations that families should take care of their elderly members. There were concerns about the impact of past negative experiences with racism and discrimination against relatives [5], low expectations around care homes adequately addressing their cultural needs such as: food [5, 23, 25, 26], following hygiene and cleanliness practices, overcoming language and communication problems [5], and lack of celebration of cultural festivals [26]. South Asian study participants emphasised the importance of better home‐based care provision for their community and family members. In contrast, there was a sense of reluctant acceptance amongst Black Caribbean people that ‘care homes are undesirable yet unavoidable part of their future’ [25] because a high proportion of Caribbean older adults in the UK live alone.

#### 4.5.2 Maintaining One's Privacy, Sense of Independence and Religious Practice

There was a strong consensus amongst African and Caribbean people that maintaining one's privacy is culturally important. Furthermore, some people in the community also associate sharing too much personal information with official agencies could risk losing their freedom [22]; therefore, maintaining a sense of control in choosing one's support packages is extremely important.

Religious beliefs and practice were an integrated part of many of the study participants' everyday life. Many individuals diagnosed with dementia used religion and spirituality as a source of comfort in coping with the dementia diagnosis [4, 18, 20]. On the other hand, there were also cases that individuals stopped attending religious services ‘for fear of doing wrong during worship’ [18]. Study participants also explained that ‘respect for religion stops you accessing (dementia) services’ [4]. They added some churches may discourage people from seeking medical help [22], and also religious leaders ‘did not engage’ in dementia discussions [20]. This suggests there may be tension or conceptual difference amongst some religious practice in accepting and understanding dementia. On the other hand, in instances where religious leaders did address dementia there were positive outcomes [20].

#### 4.5.3 Increasing Wider Community Awareness of Available Dementia Services and Encouraging Help Seeking Behaviour

A large number of studies highlighted the need to increase awareness and knowledge amongst South Asian, African and Caribbean communities regarding what dementia services are available and what support (e.g., financial) people are entitled to. One participant expressed a clear wish to assist other people in the Muslim community by developing ‘a platform for people to have their say’ and assisting in working alongside health and social care professionals [18]. Furthermore, educating healthcare professionals about cultural differences in conceptualisations of dementia and developing culturally specific guidelines were deemed important [15, 24].

### 4.6 Key Areas Highlighted During the Expert Consultation Sessions

#### 4.6.1 Consultation Session Participants

Three consultation sessions: one online focus group with South Asian supporters (*n* = 6), one in‐person focus group with African and Caribbean supporters (*n* = 6), and one in‐person family interview in Gujarati (*n* = 3) with a translator, were conducted. All participants were known to the Centre for Ethnic Health Research team and were given the lay summary report to read before the meeting. Demographics of the consultation session participants are shown in Table 5. Supplementary Material 3 in Supporting Information S1 describes the key discussion outcomes from expert consultation sessions. This section summarises the key areas that the participants highlighted in response to the initial review findings in the lay summary report.

**TABLE 5:** Expert consultation session participants demographics.

Participant	Self‐identified ethnicity	Gender	Age range	Religion	Length of time living in the U.K	Contact with dementia
1	Bengali	Female	40–49	Muslim	42 years	Supporter of someone living with dementia
2	Asian British	Female	40–49	Muslim	Since birth	Previous supporter of someone living with dementia
3	South Asian	Male	20–29	Muslim	Since birth	Supporter of someone living with dementia
4	British Asian	Male	70–79	Hindu	60 years	Supporter of someone living with dementia
5	British Indian	Male	60–69	Hindu	51 years	Supporter of someone living with dementia
6	Asian British	Female	40–49	Muslim	Since birth	Supporter of someone living with dementia
7	Somali	Female	40–49	Muslim	30 years	Supporter of someone living with dementia
8	Somali	Female	60–69	Muslim	22 years	Supporter of someone living with dementia
9	Somali	Female	40–49	Muslim	7 years	Supporter of someone living with dementia
10	Black British	Female	60–69	Christian	68 years	Supporter of someone living with dementia
11	Afro Caribbean	Female	60–69	Christian	56 years	No current direct contact with someone living with dementia
12	Black British	Female	60–69	Christian	56 years	Supporter of a friend living with dementia
13	Asian	Male	80–89	Hindu	Approx. 30 years	Person living with dementia
14	Asian	Female	80–89	Hindu	Approx. 30 years	Supporter
15	Asian	Male	60–69	Hindu	Approx. 30 years	Supporter

#### 4.6.2 Attitudes Towards, and Awareness of Dementia

There was a strong consensus amongst the participants that people in their communities who may be developing dementia symptoms are reluctant to approach mainstream dementia services in the early stage; since dementia symptoms have long been managed within their communities and families. Many acknowledged that an open discussion about mental health including dementia is not the ‘norm’ within their communities, particularly amongst older members. In some cases, this has led to limited awareness of dementia symptoms and the lack of understanding of locally available support and help.

#### 4.6.3 Supporting People in Their Community Who Have Developed Dementia Symptoms

South Asian participants highlighted the strong cultural emphasis on familial care as a moral obligation to the family. Support from immediate family members was considered crucial to African and Caribbean participants. They reported that, if support from immediate families is unavailable, community members are willing to offer their support. However, respecting people's privacy is considered particularly important in their culture, sometimes leading to people not offering their help in case this is perceived as being intrusive.

#### 4.6.4 Services and Support People Would Like in the Future

Participants shared many examples of their cultural and religious needs not being met when using mainstream health services. When seeking dementia support for their family and community members, South Asian supports seem to prefer culturally appropriate support offered in their own homes, whilst African and Caribbean supporters appeared more open to using culturally appropriate day services in the community if that can help to maintain a sense of their independence. The importance of training current and future paid carers in relation to their cultural and religious needs, as well as having consistent support from the same carer, was repeatedly highlighted as essential. Family carers acknowledged the continuous practical and mental pressure on them to care for family members with dementia but added self‐care can be a challenge as it does not always align with their traditional cultural or religious values.

## 5 Discussion

The findings from the 18 included studies and the experts‐by‐experience consultations highlight the need to develop the ‘cultural and religious essentials’ that dementia service providers should consider when working with older people from South Asian, African or Caribbean backgrounds. Dementia and mental health are still not openly discussed in many ethnic minority communities. Raising dementia awareness amongst diverse ethnic community members in a non‐stigmatising manner and ‘normalising help‐seeking’ [17] behaviour to encourage early help seeking is important, as highlighted by previous studies.

### 5.1 Recommendations for Community Organisations Supporting Older People From South Asian, African and Caribbean Communities

Provision of dementia information leaflets in various languages to ensure the information is accessible for diverse ethnic minority groups is important. However, on its own, it is not sufficient. Encouraging a wide range of ethnic minority groups to use mainstream dementia services will require not only dementia knowledge dissemination but also a cultural shift in making ‘dementia and mental health talk’ a norm for all communities. Some focus group participants have expressed hesitance to attend local peer support groups due to cultural and religious differences, but all acknowledged the benefits of sharing their challenges and connecting with other people going through similar experiences. Local charities often advertise social groups offered in South Asian languages, but more efforts could be made to connect supporters and people living with dementia from similar cultural backgrounds, for example through WhatsApp peer support groups, to cultivate a more open culture of living with dementia and to offer support to each other. Community‐based dyads‐ and family‐orientated services are crucial for South Asian and African and Caribbean families to feel they can use the mainstream dementia care services. The research team's ongoing community outreach work and review data suggests that religious leaders can help their communities by normalising talking about dementia and encourage their help‐seeking behaviour.

### 5.2 Recommendations for Health and Social Care Providers

*Inpatient care*: accessibility to culturally appropriate food and personal care were consistently highlighted as the minimum essentials to maintain their dignity by study participants. There were many examples in hospital settings that patients had no access to culturally appropriate nutritious food. For example, one hospital offered salad as Halal food. Culturally appropriate hot food should not be seen as optional or personal luxury, particularly when inpatients from diverse ethnic backgrounds are trying to recover and regain their strength.

*Home‐based care*: cultural awareness training for paid carers should include a clear ‘checklist’ of essential care components for their clients. Food preparation and personal hygiene are particularly highlighted as culturally sensitive areas. Availability of a template for co‐developing the cultural essential checklist with family members will be beneficial for paid carers, for example, buy using the Culturagram assessment framework [28]. Family carers from diverse ethnic backgrounds indicated their willingness to learn from paid carers how to provide good dementia care to their family members, but mutually collaborative working relationships need to be established first.

### 5.3 Recommendations for Policy Makers

Rather than making generic policy recommendations for ‘older people from ethnic minority backgrounds’, policy makers need to make more efforts to contact and connect with their local diverse community groups first and foremost. There is a need to develop a deeper understanding of what will be culturally acceptable and helpful dementia care packages for specific ethnic groups in their locality. When developing local policies, the involvement of people with ‘lived experience’ from specific cultures is essential to tackle mental health stigma, and to ensure acceptability and usefulness of care provision. Once the ‘cultural essentials’ are articulated by their local communities, the information could be disseminated through brief booklets that specify the cultural norms and preferences of specific communities. These booklets should be clearly signposted and be widely made available to a wide range of paid carers.

Policy makers should also commit to ensuring that ‘inclusivity’ is actionable rather than just a buzzword. Inclusivity needs to extend beyond statements such as ‘everyone is welcome’ by meaningfully highlighting differences and using language when creating policies that ensures diverse communities feel valued and respected. This is a crucial step in demonstrating that their needs are taken into account, and it requires intentionally considering diverse perspectives, experiences, and needs. This would ensure transparency about what services are provided and for whom they are provided, rather than for ‘everyone’.

### 5.4 Recommendations for Researchers

Focus group participants highlighted that many individuals from diverse ethnic communities do not see research involvement to be directly relevant to them. Those who are involved in research projects expressed their frustration of researchers not sharing their findings with them. This impacted on participants feeling research ‘being done on them’, rather than them feeling part of the active research community. Researchers should take enough time to build collaborative working relationships with diverse community members, and ensure they provide regular brief study updates to their study participants. Researchers should also spend more time ‘giving back’ to diverse communities, for example by signposting high quality local dementia services to their study participants or sharing their resources and dementia knowledge, since a large portion of diverse community members feel researchers only gather information but hardly offers anything back directly to their communities.

### 5.5 Recommendations for Community Groups Run by People From Diverse Ethnic Backgrounds

Local groups that are run by people from diverse ethnic backgrounds who are determined to tackle every day stigma attached to wider mental health issues do exist (e.g., https://www.facebook.com/EApositiveminds/). However, not many diverse ethnic community groups are easily ‘discoverable’ to a wider public through general internet searches. Some local groups seem to preserve their cultural and religious values by making their group membership specific. Our focus‐group participants explained that this is sometimes necessary for the survival of a specific community group by stopping ‘outsiders’ influencing their activities. However, local diverse ethnic groups can help dementia care providers by collaborating with local researchers, stakeholders and policy makers; so that essential care package components for specific ethnic groups which meet their cultural and religious needs are more articulated at all levels, rather than individuals having to negotiate their needs with individual care providers or when admitted to hospitals.

### 5.6 Strengths and Limitation of This Review

This narrative synthesis systematic review provides a rigorous evidence synthesis of recent original research exploring views on dementia and experiences of people from South Asian, African and Caribbean backgrounds living in the UK. Strengths of the review includes two researchers independently carrying out the quality assessment and initial analyses of the included studies. It also includes the development of recommendations through the integration of the review findings and the experts‐by‐experience knowledge obtained from consultation sessions. Even though we focussed on South Asian, African and Caribbean communities, wide heterogeneity and cultural nuances exist within these communities, so our review represents only partial views and experiences of these communities. The consultations took place in Leicester in the East Midlands; therefore, some findings may not extend/be applicable to other parts of the UK.

## 6 Conclusion

Dementia is not openly discussed or disclosed within many South Asian, African or Caribbean communities. This can lead to family carers and people with dementia feeling extremely isolated and unsupported. While increasing general dementia knowledge amongst diverse communities is crucial for encouraging help‐seeking behaviours, it is equally essential for service providers to take concrete actions to tackle stigma and incorporate their cultural and religious essentials into mainstream care and support services.

Collaborative service developments between the diverse communities, social and health care providers and policy makers are essential to ensure equitable and culturally appropriate dementia care for South Asian, African and Caribbean community members in the future.

## Conflicts of Interest

The authors declare no conflicts of interest.

## Supporting information

Supporting Information S1

## Data Availability

The data that support the findings of this study are available on request from the corresponding author. The data are not publicly available due to privacy or ethical restrictions.

## References

[1] Alzheimer’s Society (2022), https://www.alzheimers.org.uk/for-researchers/black-asian-and-minority-ethnic-communities-and-dementia-research.

[2] N. Mukadam , C. Cooper , B. Basit , and G. Livingston , “Why Do Ethnic Elders Present Later to UK Dementia Services? A Qualitative Study,” International Psychogeriatrics 23, no. 7 (September 2011): 1070–1077, 10.1017/S1041610211000214. 21349212

[3] C. Kenning , G. Daker‐White , A. Blakemore , M. Panagioti , and W. Waheed , “Barriers and Facilitators in Accessing Dementia Care by Ethnic Minority Groups: A Meta‐Synthesis of Qualitative Studies,” BMC Psychiatry 17 (December 2017): 1–3, 10.1186/s12888-017-1474-0. 28854922 PMC5577676

[4] S. Parveen , C. Peltier , and J. R. Oyebode , “Perceptions of Dementia and Use of Services in Minority Ethnic Communities: A Scoping Exercise,” Health and Social Care in the Community 25, no. 2 (March 2017): 734–742, 10.1111/hsc.12363. 27278859

[5] M. Z. Hossain and H. T. Khan , “Barriers to Access and Ways to Improve Dementia Services for a Minority Ethnic Group in England,” Journal of Evaluation in Clinical Practice 26, no. 6 (December 2020): 1629–1637, 10.1111/jep.13361. 32022982

[6] J. Hailstone , N. Mukadam , T. Owen , C. Cooper , and G. Livingston , “The Development of Attitudes of People From Ethnic Minorities to Help‐Seeking for Dementia (APEND): A Questionnaire to Measure Attitudes to Help‐Seeking for Dementia in People From South Asian Backgrounds in the UK,” International Journal of Geriatric Psychiatry 32, no. 3 (March 2017): 288–296, 10.1002/gps.4462. 27001896

[7] ONS, https://www.ethnicity-facts-figures.service.gov.uk/uk-population-by-ethnicity/national-and-regional-populations/population-of-england-and-wales/latest.

[8] J. Popay , H. Roberts , A. Sowden , M. Petticrew , et al. “Guidance on the Conduct of Narrative Synthesis in Systematic Reviews,” A Product From the ESRC Methods Programme Version 1 (2006): b92.

[9] O. McDermott , N. Crellin , H. M. Ridder , and M. Orrell , “Music Therapy in Dementia: A Narrative Synthesis Systematic Review,” International Journal of Geriatric Psychiatry 28, no. 8 (August 2013): 781–794, 10.1002/gps.3895. 23080214

[10] M. J. Page , J. E. McKenzie , P. M. Bossuyt , et al. “The PRISMA 2020 Statement: An Updated Guideline for Reporting Systematic Reviews,” BMJ 372 (March 2021): n71, 10.1136/bmj.n71. 33782057 PMC8005924

[11] Critical Appraisal Skills Programme , CASP Qualitative Studies Checklist, (2022), https://casp-uk.net/casp-tools-checklists/.

[12] A. Tong , P. Sainsbury , and J. Craig , “Consolidated Criteria for Reporting Qualitative Research (COREQ): A 32‐Item Checklist for Interviews and Focus Groups,” International Journal for Quality in Health Care 19, no. 6 (December 2007): 349–357, 10.1093/intqhc/mzm042. 17872937

[13] M. Z. Hossain , S. A. Tarafdar , T. Kingstone , P. Campbell , and C. A. Chew‐Graham , “From Detection to Preparing for the End‐Of‐Life: A Qualitative Exploration of the South Asian Family Carers' Experiences of the Journey With Dementia,” Health and Social Care in the Community 30, no. 6 (November 2022): e5135–e5144, 10.1111/hsc.13930. 35906825

[14] M. Z. Hossain and H. T. Khan , “Dementia in the Bangladeshi Diaspora in England: A Qualitative Study of the Myths and Stigmas About Dementia,” Journal of Evaluation in Clinical Practice 25, no. 5 (October 2019): 769–778, 10.1111/jep.13117. 30811845

[15] J. La Fontaine , J. Ahuja , N. M. Bradbury , S. Phillips , and J. R. Oyebode , “Understanding Dementia Amongst People in Minority Ethnic and Cultural Groups,” Journal of Advanced Nursing 60, no. 6 (December 2007): 605–614, 10.1111/j.1365-2648.2007.04444.x. 18039247

[16] J. Mackenzie , “Stigma and Dementia: East European and South Asian Family Carers Negotiating Stigma in the UK,” Dementia 5, no. 2 (May 2006): 233–247, 10.1177/1471301206062252.

[17] N. Mukadam , A. Waugh , C. Cooper , and G. Livingston , “What Would Encourage Help‐Seeking for Memory Problems Among UK‐Based South Asians? A Qualitative Study,” BMJ Open 5, no. 9 (September 2015): e007990, 10.1136/bmjopen-2015-007990. PMC4567682 26362662

[18] J. L. Regan , “Ethnic Minority, Young Onset, Rare Dementia Type, Depression: A Case Study of a Muslim Male Accessing UK Dementia Health and Social Care Services,” Dementia 15, no. 4 (July 2016): 702–720, 10.1177/1471301214534423. 24858552

[19] T. James , N. Mukadam , A. Sommerlad , S. Barrera‐Caballero , and G. Livingston , “Equity in Care and Support Provision for People Affected by Dementia: Experiences of People From UK South Asian and White British Backgrounds,” International Psychogeriatrics 36, no. 7 (February 2023): 564–573, 10.1017/s1041610223000121. 36803586

[20] P. Kevern , D. Lawrence , N. Nazir , and A. Tsaroucha , “Religious Influences on the Experience of Family Carers of People With Dementia in a British Pakistani Muslim Community,” InHealthcare 11, no. 1 (December 2022): 120: MDPI, 10.3390/healthcare11010120. PMC9819143 36611580

[21] N. Nazir and P. Kevern , “Understanding and Awareness of Dementia in the Pakistani‐Origin Community of Stoke‐On‐Trent, UK: A Scenario‐Based Interview Study,” InHealthcare 12, no. 2 (January 2024): 251: MDPI, 10.3390/healthcare12020251. PMC10815018 38275532

[22] S. Berwald , M. Roche , S. Adelman , N. Mukadam , and G. Livingston , “Black African and Caribbean British Communities’ Perceptions of Memory problems: ‘We Don’t Do dementia.’,” PLoS One 11, no. 4 (April 2016): e0151878, 10.1371/journal.pone.0151878. 27045999 PMC4821595

[23] S. Baghirathan , R. Cheston , R. Hui , A. Chacon , P. Shears , and K. Currie , “A Grounded Theory Analysis of the Experiences of Carers for People Living With Dementia From Three BAME Communities: Balancing the Need for Support Against Fears of Being Diminished,” Dementia 19, no. 5 (July 2020): 1672–1691, 10.1177/1471301218804714. 30318901

[24] S. Brown , G. Livingston , and N. Mukadam , “A National Memory Clinic Survey to Assess Provision for People From Diverse Ethnic Backgrounds in England and Wales,” International Journal of Environmental Research and Public Health 18, no. 4 (February 2021): 1456, 10.3390/ijerph18041456. 33557171 PMC7913949

[25] V Lawrence , J Murray , K Samsi , and S Banerjee , “Attitudes and support Needs of Black Caribbean, south Asian and White British Carers of People With Dementia in the UK,” British Journal of Psychiatry 193, no. 3 (September 2008): 240–246, 10.1192/bjp.bp.107.045187. 18757985

[26] V Lawrence , K Samsi , S Banerjee , C Morgan , and J Murray , “Threat to Valued Elements of Life: The Experience of Dementia Across Three Ethnic Groups,” Gerontologist 51, no. 1 (February 2011): 39–50, 10.1093/geront/gnq073. 20724657

[27] P Nair , Y Barrado‐Martín , K Anantapong , K Moore , C Smith , E Sampson , J Manthorpe , K Walters , and N Davies , “Experiences of Carers and People With Dementia From Ethnic Minority Groups Managing Eating and Drinking at Home in the United Kingdom,” Nutrients 14, no. 12 (June 2022): 2395, 10.3390/nu14122395. 35745124 PMC9230659

[28] EP Congress , “The Use of Culturagrams to Assess and EmpowerCulturally Diverse Families,” Families in Society 75, no. 9 (November 1994): 531–540, 10.1177/1044389494075009.

