# Supplementary material for: What Works Well for People With Dementia and Their Supporters From South Asian, African and Caribbean Communities in the UK: A Narrative Synthesis Systematic Review and Expert Consultations
Source: Int J Geriatr Psychiatry. 2025 Feb 26;40(3):e70047. doi: 10.1002/gps.70047 (PMC11864919; doi:10.1002/gps.70047)
Supplement: Supplementary file 1 — Supporting Information S1 [file GPS-40-e70047-s001.docx]

**Supplementary Material 1 Search Strategies**

**Search Methods:**

The Information Specialist searched the following resources up to 20 December 2022 to identify relevant papers:

- the Cochrane Central Register of Controlled Trials (CENTRAL) Issue 11, 2022, in the Cochrane Library;
- MEDLINE Ovid (from 1946 onwards);
- APA PsycInfo ProQuest (from 1806 onwards);
- APA PsycArticles ProQuest (from 1800 onwards);
- Web of Science (Clarivate) (from 1900 onwards);
- Scopus Elsevier (from 1788 onwards).

| **Platform and database**: Ovid Medline® ALL <1946 to 20 December 2022> | | |
| --- | --- | --- |
|  |  |  |
| 1 | dementia/ or alzheimer disease/ | 165055 |
| 2 | (dement* or alzheimer*).tw,kw,kf. | 268099 |
| 3 | 1 or 2 | 286022 |
| 4 | "Ethnic and Racial Minorities"/ | 437 |
| 5 | racial groups/ or blacks/ or asians/ or ethnicity/ | 193498 |
| 6 | Minority Groups/ or Minority Health/ or Cultural Diversity/ | 29152 |
| 7 | (black*1 or asian*1 or BAME or race or racial or ethnic* or minorit* or multi?ethnic* or multi?racial or multi?cultur* or BME or "people of colo?r" or "person of colo?r" or bengali* or indian* or chinese or pakistani* or african* or gypsy or gypsies or irish traveller* or roma or arab*1 or "afro?caribbean*" or afrocaribbean*).tw,kw,kf. | 1058915 |
| 8 | or/4-7 | 1134786 |
| 9 | health services/ or community health services/ or preventive health services/ or Social Work/ | 88446 |
| 10 | ((access*2 or accessing or accessibility or deliver* or provision or provid*3 or quality or equit* or disparit*) adj4 (Service* or clinic*1 or healthcare or health?care or social care)).tw,kw,kf. | 219015 |
| 11 | health services accessibility/ or "delivery of health care"/ or health equity/ or healthcare disparities/ or quality of healthcare/ or Cross-Cultural Comparison/ or Health Knowledge, Attitudes, Practice/ or Communication Barriers/ | 422672 |
| 12 | or/9-11 | 657416 |
| 13 | 3 and 8 and 12 | **823** |
|  |  |  |
| **Platform and database**: CENTRAL Issue 11 2022, Cochrane Library | | |
|  |  |  |
| #1 | MeSH descriptor: [Dementia] this term only | 2858 |
| #2 | MeSH descriptor: [Alzheimer Disease] this term only | 3866 |
| #3 | (dement* or alzheimer*):ti,ab | 22087 |
| #4 | {OR #1-#3} | 22527 |
| #5 | MeSH descriptor: [Ethnic and Racial Minorities] this term only | 9 |
| #6 | MeSH descriptor: [Racial Groups] this term only | 503 |
| #7 | MeSH descriptor: [Blacks] this term only | 844 |
| #8 | MeSH descriptor: [Asians] this term only | 2443 |
| #9 | MeSH descriptor: [Ethnicity] this term only | 960 |
| #10 | MeSH descriptor: [Minority Groups] this term only | 414 |
| #11 | MeSH descriptor: [Minority Health] this term only | 26 |
| #12 | MeSH descriptor: [Cultural Diversity] this term only | 76 |
| #13 | (black* or asian* or BAME or race or racial or ethnic* or minorit* or multi?ethnic* or multi?racial or multi?cultur* or BME or "people of colo?r" or "person of colo?r" or bengali* or indian* or chinese or pakistani* or african* or gypsy or gypsies or "irish traveller*" or roma or arab* or "afro?caribbean*" or afrocaribbean*):ti,ab | 81851 |
| #14 | {OR #5-#13} | 83371 |
| #15 | MeSH descriptor: [Health Services] this term only | 492 |
| #16 | MeSH descriptor: [Community Health Services] this term only | 1095 |
| #17 | MeSH descriptor: [Preventive Health Services] this term only | 533 |
| #18 | MeSH descriptor: [Social Work] this term only | 187 |
| #19 | ((access* or deliver* or provision or provid* or quality or equit* or disparit*) NEAR/4 (Service* or clinic* or healthcare or health?care or social care)):ti,ab | 57288 |
| #20 | MeSH descriptor: [Health Services Accessibility] this term only | 750 |
| #21 | MeSH descriptor: [Delivery of Health Care] this term only | 956 |
| #22 | MeSH descriptor: [Health Equity] explode all trees | 10 |
| #23 | MeSH descriptor: [Healthcare Disparities] explode all trees | 206 |
| #24 | MeSH descriptor: [Quality of Health Care] this term only | 926 |
| #25 | MeSH descriptor: [Cross-Cultural Comparison] explode all trees | 232 |
| #26 | MeSH descriptor: [Health Knowledge, Attitudes, Practice] this term only | 6350 |
| #27 | MeSH descriptor: [Communication Barriers] explode all trees | 114 |
| #28 | {OR #15-#27} | 65933 |
| #29 | #4 and #14 and #28 | **135 (134)** |
|  |  |  |
| **Platform and database**: Scopus Elsevier | | |
|  |  |  |
| 1 | TITLE-ABS((access* or deliver* or provision or provid* or quality or equity or equities or disparit*) W/4 (Service* or clinic* or healthcare or "health care" or "social care")) |  |
| 2 | TITLE(dement* OR alzheimer*) |  |
| 3 | TITLE(black* or asian* or BAME or race or racial or ethnic* or minorit* or "multi ethnic*" or "multi racial" or "multi cultur*" or BME or "people of color" OR "people of colour" OR "person of color" OR "person of colour" or bengali* or indian* or chinese or pakistani* or african* or gypsy or gypsies or "irish traveller*" or roma or arab* or "afro caribbean*" or afrocaribbean*) |  |
| 4 | 1 AND 2 AND 3 | **172** |
|  |  |  |
| **Platform and database**: ProQuest APA PsycInfo® and PsycArticles | | |
|  | PsycINFO |  |
| S1 | (MAINSUBJECT.EXACT("Ethnic Identity") OR (MAINSUBJECT.EXACT("Blacks") OR MAINSUBJECT.EXACT("Minority Groups")) OR MAINSUBJECT.EXACT("Racial and Ethnic Groups") OR MAINSUBJECT.EXACT("Asians") OR TI,AB(black* OR asian* OR BAME OR race OR racial OR ethnic* OR minorit* OR multi?ethnic* OR multi?racial OR multi?cultur* OR BME OR "people of colo?r" OR "person of colo?r" OR bengali* OR indian* OR chinese OR pakistani* OR african* OR gypsy OR gypsies OR irish traveller* OR roma OR arab* OR "afro?caribbean*" OR afrocaribbean*)) AND (MAINSUBJECT.EXACT("Alzheimer's Disease") OR MAINSUBJECT.EXACT("Dementia") OR TI,AB(dement* OR alzheimer*)) AND (((MAINSUBJECT.EXACT("Community Mental Health Services") OR MAINSUBJECT.EXACT("Preventive Health Services")) OR MAINSUBJECT.EXACT("Health Disparities")) OR (MAINSUBJECT.EXACT("Cross Cultural Treatment") OR (MAINSUBJECT.EXACT("Cultural Sensitivity") OR MAINSUBJECT.EXACT("Cross Cultural Communication") OR MAINSUBJECT.EXACT("Cross Cultural Differences"))) OR (tiab((service OR services* OR clinic OR clinics OR healthcare OR "health care" OR "social care")) NEAR/4 tiab((access* OR delivery OR provision OR provid* OR quality OR equity OR equities OR disparit*)))) | **499** |
|  |  |  |
|  | PsycArticles – strategy as above | **7** |
|  |  |  |
| **Platform and database**: Web of Science (Clarivate) | | |
|  |  |  |
|  | Query link: <https://www.webofscience.com/wos/woscc/summary/a7fce9f6-e475-447b-bd76-a1a589f7cc68-668295ff/relevance/1> |  |
| 1 | dement* OR alzheimer* (Title) | 178823 |
| 2 | (black* or asian* or BAME or race or racial or ethnic* or minorit* or "multi ethnic*" or "multi racial" or "multi cultur*" or BME or "people of color" OR "people of colour" OR "person of color" OR "person of colour" or bengali* or indian* or chinese or pakistani* or african* or gypsy or gypsies or "irish traveller*" or roma or arab* or "afro caribbean*" or afrocaribbean*) (Title) | 1178148 |
| 3 | TI=(((access* or deliver* or provision or provid* or quality or equity or equities or disparit*) NEAR/4 (Service* or clinic* or healthcare or "health care" or "social care"))) | 82286 |
| 4 | AB=(((access* or deliver* or provision or provid* or quality or equity or equities or disparit*) NEAR/4 (Service* or clinic* or healthcare or "health care" or "social care"))) | 649678 |
| 5 | #3 OR #4 | 689610 |
| 6 | #1 AND #2 AND #5 | **145** |
|  |  |  |

**Supplementary Material 2. Quality Assessment**

The CASP consists of 10 questions, with 2 items related to screening and 8 items related to appraisal. Although the CASP is popular due to its ease of use and administration and its comprehensibility (Majid & Vanstone, 2018)​, it may not be the strongest tool for evaluating methodological quality compared to other appraisal tools ​(Hannes et al., 2010). To address the limitations of the CASP Checklist in evaluating methodological quality, a modified version of the Consolidated Criteria for Reporting Qualitative Studies (COREQ) was additionally used.

The COREQ Checklist consists of 32 structured statements across three domains: research team and reflexivity, study design, and analysis and findings ​(Tong et al., 2007). However, for this review, only the domains of *study design* and *analysis of findings* were utilised in the modified versions, which were tailored for both qualitative and mixed methods studies. The checklist was condensed to 12 items for qualitative studies and 11 items for mixed methods studies.

Qualitative studies were assessed using: a) the CASP Checklist, utilising the following CASP scoring items: “Yes” (1); “No” (0); “Can’t tell” (0); “Valuable” (1), and b) the Modified COREQ Checklist for qualitative studies. Mixed-method studies were assessed using the Modified COREQ Checklist for mixed-method studies. Both modified versions of the COREQ checklist were scored as follows: “Fully met” (2); “Partially met” (1); “Can’t tell (0); “No” (0).

A minimum CASP score of 7 out of 10, and a minimum Modified COREQ score of 10 out of 24 (qualitative studies) and 22 (mixed-method) were required for a study to be included in this review.

**Supplementary Material 3: Key discussion outcomes from expert consultation sessions**

***Attitudes towards, and awareness of dementia***

All participants who are current supporters of someone living with dementia, clearly expressed their determination to provide high quality care to support their relatives living with dementia. Their commitment and dedication to take care of older members of their direct and extended family members, which are deeply rooted in their cultural and religious upbrings, was evident in all consultation sessions. The participants have acknowledged the physical and emotional impacts of being a supporter of someone living with dementia. However, many acknowledged talking about dementia and associated challenges openly was not ‘the norm’ amongst their communities:

‘Very difficult when it comes to dementia, where this is mental – there’s a lot of stigma attached, so people are not gonna talk about it…. With dementia, within mental health, we found people stay away. They give the distance’ (P3)

Further comments confirmed what have been highlighted by the included studies:

*Respecting another person’s privacy:*

‘I don’t want to interfere *(with her friend who may be developing dementia)* because she’s got family members – she’s in denial, but I’m not a family member and I can’t just like….’. (P12)

*Lack of knowledge of dementia as a health condition:*

‘Even as a nurse, you weren’t educated into the finer knowledge of the condition’. (P4)

‘Lack of knowledge, if you don’t understand something, you’re going to interpret it any way you want’.

***Supporting people in their community who have developed dementia symptoms***

*Experiences and perspectives of South Asian participants*

As highlighted by the literature, the commitment of younger members to provide direct care and support to their older family members at home was evident in all consultation sessions, as articulated by one participant: ‘most of us are seeking that kind of support or best practice for our relatives’. Psychological and physical impact of providing continuous care for a close family member with dementia was evident, even when the participants insisted that they were willingly providing care to their relatives. Retired family carers (e.g., spouse) tried to maintain at least one activity that the individual found psychologically or spiritually meaningful (e.g. Yoga session) but stated going to local dementia support groups would not be meaningful due to the cultural differences and the language barriers for older carers. South Asian carers were open to ideas of attending culturally appropriate peer support groups to share their experiences but highlighted the practical challenge of finding an appropriate support group.

Difficulties of being a South Asian inpatient in a hospital setting were highlighted by several participants. The lack of culturally and religiously appropriate cooked food, communication difficulties for South Asian elders due to the language barriers, and the lack of culturally appropriate personal care were repeatedly discussed in the group. Relatives acknowledged the staff shortage and the challenge of hospital set-up to provide culturally appropriate care for all patients, but the group clearly expressed their frustration: ‘‘How do we make people better by accessing services that are not designed for us? How not exacerbating the condition *(by not being providing appropriate food or not being to speak their own language)*, especially with dementia” (P3)

When asked whether they would consider using the external mainstream dementia support services, some shared they already use respite and home-visit services. but also highlighted the challenge of finding a consistent carer who understands their cultural and religious needs. Complexity of navigating the funding and application process was also highlighted, many emphasising the importance of persistence.

*Experiences and perspectives of African and Caribbean participants*

Three focus group members were Christian and had lived in the UK for most of their life, while other three members were Muslim who migrated from Somalia. The Somali participants explained their cultural context of caring:

“…culturally it is a stigma if a family member takes their parents or grandparents to a care centre. So, you have to take care of them, no matter how hard it is, whether you and your children or whether you and your husband or the family, extended family. So that is how we've been brought up. Otherwise, you'll be alienated from the whole community.” (P7)

Within the community, maintaining dignity and respect for older members were considered as particularly important: ‘It’s about making them feel important…. independence is a big deal to a lot of them, and they don’t want to feel as though they’re being pitied’. (P9 and P7)

The research team asked how supporters take care of themselves to keep going while supporting their family members with dementia. One participant immediately responded by saying ‘I feel emotional’ (P8). A fellow participant, who shares their cultural and religious backgrounds, explained: ‘it’s not in our culture or religion… we've not been brought up to do that…. now you hear about self-love and self-care and all of that, which is beautiful and brilliant, but it's just we give and give and give to the point where you can’t pull from any pot’ (P8). Whilst the participants agreed on the challenge of navigating through traditional values, all agreed the importance of taking care of ‘ourselves’ when supporting someone living with dementia.

One participant, who is supporting her friend with dementia, echoed one of the challenges highlighted in the literature: ‘…my concern is how do you get the help for those people who’ve not got immediate family around them?’ (P12). She explained ‘grown-up children’ of her friends and community members often move away and have their own life elsewhere while aging parents may live alone, voicing a common issue in the UK.

***Services and support people would like in the future***

There was a strong consensus in both South Asian and African & Caribbean groups that culturally appropriate community-based family support packages would work well. In line with the literature review findings, a number of our focus group participants stated that understanding and navigating through the UK funding and support scheme for people with dementia was a challenge and required supporters to be self-resilient and ‘pushy’ at times. South Asian supporters seem to prefer culturally appropriate external support offered in their own homes, whilst African and Caribbean supporters seemed more open to ideas of using culturally appropriate community-based services such as day centres. When agency (external) carers were providing extra support in their homes, the focus group participants emphasised with the time pressure of individual carers who need to cover a number of homes in a limited time. At the same time, they highlighted the fundamental shortfalls of these external agency carer visits, such as: ‘not contacting you when they are late’ (P9 & P15) and not being able to book the same carer who knows their family member with dementia. The participants highlighted the importance of an external carer working collaboratively with family carers: “If they're coming into that person's home, every home is going to be different. But that carer needs to work alongside a family member and saying this is how the person likes this done, wants this done, makes this person happy.” (P1) One participant stated: ‘(an external carer) taught me how to do it (i.e., how to provide practical care to a family member)’’ (P3), highlighting that family members are willing to learn skills from external carers.

Educating wider members of their communities about dementia, including their children, was considered as essential by many participants. The participants also linked the importance of active involvement in dementia research:

*“I'm contributing towards the future that you, as researchers, are going to take some of my words and actually be able to write a report about something like that. And actually my story will be told. People will understand that actually, as people, we contributed towards making things better. And I think we all have our part to play.”* (P3)

*‘this (taking part in research) is something new to our (Somali) community, because before, and even me as a person, I didn't participate in any research, because a lot of the time, people would say, what's the point? A little time, but we forget that straw that broke the camel’s back, all of this information or all of that research, all of that one day, if not our generation, the next generation will benefit. So, for us to go out there, to participate, to work with Barbara and the team, to be able to work with other universities, to open up. It really meant a lot, and it's really needed’ (P9)*

Participants also openly shared their experience of discrimination and racism, including receiving stereotypical comments, that they experienced while taking care of relatives in hospitals or in the community. This highlights the importance of co-developing the future service provision strategies and policies for ‘people with dementia from ethnic minority groups’, so that stereotypical assumptions of their preference could be checked by relevant community members with lived experience.
